# Analysis of multiple gene co-expression networks to discover interactions favoring CFTR biogenesis and ΔF508-CFTR rescue

**DOI:** 10.1186/s12920-021-01106-7

**Published:** 2021-10-30

**Authors:** Matthew D. Strub, Long Gao, Kai Tan, Paul B. McCray

**Affiliations:** 1 Department of Pediatrics, University of Iowa, 6320 PBDB, 169 Newton Road, Iowa City, IA 52242 USA; 2 Interdisciplinary Graduate Program in Genetics, University of Iowa, Iowa City, IA 52245 USA; 3 Department of Genetics, University of Pennsylvania, Philadelphia, PA 19104 USA; 4 Center for Childhood Cancer Research, Children’s Hospital of Philadelphia, Philadelphia, PA 19104 USA; 5 Department of Pediatrics, University of Pennsylvania, Philadelphia, PA 19104 USA

**Keywords:** Cystic fibrosis, CFTR, Transcriptional profiling, Network biology, Gene modules, Gene ontology, M-module

## Abstract

**Background:**

We previously reported that expression of a miR-138 mimic or knockdown of *SIN3A* in primary cultures of cystic fibrosis (CF) airway epithelia increased ΔF508-CFTR mRNA and protein levels, and partially restored CFTR-dependent chloride transport. Global mRNA transcript profiling in ΔF508-CFBE cells treated with miR-138 mimic or *SIN3A* siRNA identified two genes, *SYVN1* and *NEDD8*, whose inhibition significantly increased ΔF508-CFTR trafficking, maturation, and function. Little is known regarding the dynamic changes in the CFTR gene network during such rescue events. We hypothesized that analysis of condition-specific gene networks from transcriptomic data characterizing ΔF508-CFTR rescue could help identify dynamic gene modules associated with CFTR biogenesis.

**Methods:**

We applied a computational method, termed M-module, to analyze multiple gene networks, each of which exhibited differential activity compared to a baseline condition. In doing so, we identified both unique and shared gene pathways across multiple differential networks. To construct differential networks, gene expression data from CFBE cells were divided into three groups: (1) siRNA inhibition of *NEDD8* and *SYVN1*; (2) miR-138 mimic and *SIN3A* siRNA; and (3) temperature (27 °C for 24 h, 40 °C for 24 h, and 27 °C for 24 h followed by 40 °C for 24 h).

**Results:**

Interrogation of individual networks (e.g., NEDD8/SYVN1 network), combinations of two networks (e.g., NEDD8/SYVN1 + temperature networks), and all three networks yielded sets of 1-modules, 2-modules, and 3-modules, respectively. Gene ontology analysis revealed significant enrichment of dynamic modules in pathways including translation, protein metabolic/catabolic processes, protein complex assembly, and endocytosis. Candidate CFTR effectors identified in the analysis included *CHURC1, GZF1,* and *RPL15,* and siRNA-mediated knockdown of these genes partially restored CFTR-dependent transepithelial chloride current to ΔF508-CFBE cells.

**Conclusions:**

The ability of the M-module to identify dynamic modules involved in ΔF508 rescue provides a novel approach for studying CFTR biogenesis and identifying candidate suppressors of ΔF508.

**Supplementary Information:**

The online version contains supplementary material available at 10.1186/s12920-021-01106-7.

## Introduction

Cystic fibrosis (CF) is the most common lethal autosomal disease in Caucasian populations, affecting approximately 75,000 individuals worldwide [1, 2]. CF harms multiple organ systems and can cause meconium ileus, growth failure, diabetes, increased sweat chloride concentrations, and infertility, among other symptoms [3–10]. However, the majority of CF-associated morbidity and mortality results from chronic and progressive lung dysfunction, characterized by acidic airway surface liquid, weakened antimicrobial defenses at the airway surface, and impaired mucociliary transport leading to chronic bacterial airway infections, irreversible tissue remodeling, and respiratory failure.

CF is caused by mutations in *cystic fibrosis transmembrane conductance regulator* (*CFTR*) which encodes an anion channel. Although over 2000 mutations have been identified in *CFTR*, over 70% of disease-associated alleles contain a deletion of phenylalanine at position 508, termed ΔF508, correlating to roughly 90% of individuals with CF having one or two ΔF508 alleles [11–15]. The ΔF508-CFTR mutation results in protein misfolding and proteasomal degradation [16, 17]. The observation that low temperature (27 °C) incubation could rescue and traffic ΔF508-CFTR to the cell surface was compelling because it demonstrated that partial function could be retained if ΔF508-CFTR escaped the endoplasmic reticulum-associated degradation (ERAD) pathway and trafficked to the cell membrane [16]. Although manipulation of temperature is not therapeutically practical, this observation encouraged the investigation of genes affecting the processing and maturation of CFTR. We previously identified microRNA-138, *SIN3A, NEDD8*, and *SYVN1* as members of the CFTR biogenesis pathway [18, 19]. Functional assays determined that miR-138 overexpression or siRNA knockdown of *SIN3A, SYVN1,* or *NEDD8* partially restored the maturation, trafficking, and function of ΔF508-CFTR.

Additional transcriptomic studies have identified effectors of CFTR biogenesis. For example, Clarke and colleagues performed a microarray study of primary epithelial cells from ΔF508 homozygotes and non-CF controls, which yielded a molecular signature of native CF airway epithelial cells in which a noteworthy number of genes involved in inflammation and defense were upregulated, including *S100A8*, *S100A9*, and *SERPINA3* [20]*.* A follow-up meta-analysis by Clark et al. identified several negative regulators of CFTR, including *SNX6, PSEN1,* and *RCN2* [21]. When these genes were knocked down via siRNA, a considerable increase in CFTR trafficking to the cell membrane was observed.

Despite the identification of several in vitro effectors of ΔF508-CFTR correction, its biogenesis pathway remains incompletely understood. Analyzing and understanding rescue-specific molecular events are critical for understanding CF biogenesis and the development of therapeutics. Network biology is a powerful tool for analyzing such complex disease phenotypes. For example, Taylor et al. demonstrated that hub gene topology could be used to improve the prognosis of breast cancer, while Chuang and colleagues revealed that differentially expressed subnetworks are effective biomarkers for breast cancer metastasis [22, 23]. However, a common theme of network biology is the dichotomization of disease progression, either for the onset or severity of disease, as many studies analyze each condition individually. Such studies highlight hubs, modules, or edges that are significantly associated with only a single condition, rather than modeling the gene expression data as a single continuum. This ultimately limits our ability to observe changes at a pathway level during disease progression.

Likewise, focusing on only CF vs. non-CF omics data, or the analysis of only a single rescue signature, may limit the detection of CFTR biogenesis interactors. To address this critical gap in network biology, Tan and colleagues developed a general framework, termed M-module, to reveal subnetwork dynamics by joint analysis of multiple gene co-expression networks [24, 25]. They demonstrated that the use of network connectivity dynamics significantly improved the classification accuracy of multiple breast cancer stages. Here, we use this M-module framework to analyze multiple gene co-expression networks relevant to CFTR to identify interactions favoring CFTR biogenesis (Table 1). These networks include miR-138 overexpression and *SIN3A* knockdown, *NEDD8* and *SYVN1* knockdown, and low temperature treatments.

**Table 1:** Co-expression networks analyzed using the M-module framework

Group	Control	Treatments
Temperature	37 °C	27 °C for 24 h, 40 °C for 24 h, 27 °C for 24 h followed by 40 °C for 24 h
NEDD8	Scrambled siRNA	siRNA inhibition of *NEDD8* and *SYVN1*
miR-138	Scrambled siRNA	miR-138 mimic and siRNA inhibition of *SIN3A*

## Materials and methods

### M-module analysis

To identify both unique and shared gene pathways across multiple differential networks, we grouped gene expression data from CFBE41o^−^ cells (GSE142610) into three conditions (Table 1). The first group, termed “miR-138/SIN3A”, compared overexpression of miR-138 and siRNA knockdown of *SIN3A* with a scrambled siRNA control [18]. The second group, termed “NEDD8/SYVN1”, compared siRNA knockdown of *NEDD8* and *SYVN1* compared to a scrambled siRNA control [19]. The third group, termed “Temperature”, included treatment conditions of 27 °C for 24 h, 40 °C for 24 h, and 27 °C for 24 h followed by 40 °C for 24 h, compared to a 37 °C control [16]. A summary and full lists of differentially expressed genes across conditions can be found in Additional file 1: Tables S1-S4, and Figure S1. We used a literature-based curated list of 333 CFTR-associated genes, termed the “CFTR Interactome”, as seed nodes (Additional file 1: Table S5). The gene expression profiles across the multiple rescue conditions were superimposed on human protein–protein interaction networks and used to build differential gene networks [26–29]. First, we constructed a binary expression network with edges chosen based on the absolute value of Pearson correlation of the expression profiles of two genes (***i,j***). Edges whose correlation did not exceed a pre-defined threshold ***δ*** were removed from the binary network. Remaining edges were then weighted (***w***_***i,j***_) using the geometric mean of the *p* values (***p***_***i***_ and ***p***_***j***_) of differential gene expression between the baseline and rescue conditions. Second, multiple differential networks were analyzed to identify multiple differential modules (M-DMs) under different conditions. 1-Modules are modules that were only found in one experimental condition whereas M-Modules with M ≥ 2 are modules that were found in multiple conditions.

### Module gene significance

Module gene significance was determined by running 10,000 randomized iterations of the M-module analysis and calculating average distances of non-seed genes to seed genes. If the distance was significantly less in the randomized networks, then the gene was considered insignificant in our module output. Genes with FDR < 0.01 were considered significant.

### Gene ontology enrichment analysis

We next ranked non-seed significant module genes by minimum distance to closest seed genes. Gene Ontology (GO) enrichment analysis was performed on the non-seed significant module genes. For each enriched GO term, the average minimum distance between contributing non-seed module genes and CFTR-associated seed genes was calculated. Such analyses allowed for the unbiased identification of candidate genes potentially involved in CFTR-relevant pathways and functions.

### Cultured cells

CFBE41o^−^ cells, termed “CFBE cells”, originally developed by immortalization of CF airway epithelial cells and later transduced with a ΔF508-CFTR expression cassette using the TranzVector lentivirus system were cultured as previously described [30–32]. These cells were obtained from Dr. J.P. Clancy at Cincinnati Children’s Hospital.

### Oligonucleotide reagents

Dicer-Substrate Short Interfering RNAs (DsiRNAs) were obtained as TriFECTa kits from IDT (Coralville, IA), each containing three pre-designed siRNAs per gene. Transfections were performed in 24-well plates, with 3 μL of Lipofectamine RNAiMAX (Invitrogen, Carlsbad, CA) and 1 μL of each 10 μM DsiRNA added to 94 μL of Opti-MEM (Gibco, Waltham, MA) media per well. Following a five minute incubation at room temperature, 50 μL of the DsiRNA-lipid complex was added to each well and 200s cells were suspended in 100 μL of Opti-MEM and seeded onto 24-well plates. Knockdown efficiency was measured following 24 h of incubation at 37 °C. To ascertain the specificity of the oligonucleotides, we harvested RNA from cells transfected with the three pooled oligonucleotides per gene and measured the expression of multiple genes 24 h post-transfection. First-strand cDNA was synthesized using SuperScript II (Invitrogen, Carlsbad, CA) with oligo-dT and random-hexamer primers. Primers for each gene were designed and produced by IDT and validated in HEK cells. Quantitative RT-PCR was performed using the QuantStudio 6 Flex Real-Time PCR system (Applied Biosystems, Foster City, CA). All experiments were performed in quadruplicate. Following validation of knockdown efficiency, three siRNAs per gene were pooled and CFBE cells were reverse-transfected and grown on microporous membranes of Transwell (Corning, Corning, NY) plates seven days prior to the electrophysiology measurements [33]. Oligo sequences and siRNA knockdown efficiencies can be found in Additional file 1: Table S6.

### Transepithelial chloride current studies

Transepithelial chloride current measurements were made in Ussing chambers approximately seven days post-seeding. Briefly, epithelial sheets were mounted in the Ussing chamber and transepithelial chloride current (I_t_) was measured under short-circuit conditions. After measuring baseline current, 100 μM amiloride (Amil) was added apically to inhibit epithelial sodium channels (ENaC) followed by apical addition of 100 μM 4,4′-diisothiocyanoto-stilbene-2,2′-disulfonic acid (DIDS) to inhibit non-CFTR chloride channels. Next, we applied 10 μM forskolin and 100 μM 3-isobutyl-1-methylxanthine (IBMX). These agents elevate intracellular cAMP levels leading to the PKA-mediated phosphorylation and activation of CFTR channels. Finally, 100 μM GlyH-101, an inhibitor of CFTR channels, was added apically. Studies were conducted with 135 mM NaCl, 1.2 mM MgCl_2_, 1.2 mM CaCl_2_, 2.4 mM K_2_PO_4_, 0.6 mM KH_2_PO_4_, 5 mM dextrose, and 5 mM HEPES (pH 7.4) on the basolateral surface, and an apical chloride concentration gradient with gluconate substituted for chloride.

### Statistical analysis

For electrophysiology studies, the average change in peak transepithelial current (I_t_) was calculated and statistical significance was determined using the Brown-Forsythe ANOVA with Benjamini–Hochberg multiple comparison correction (*p < 0.05) [34]. Data are presented as a mean ± standard error of individual data points. *p* < 0.05 was considered significant.

## Results

### Identification of individual rescue condition-specific modules and networks

**Fig. 1:**
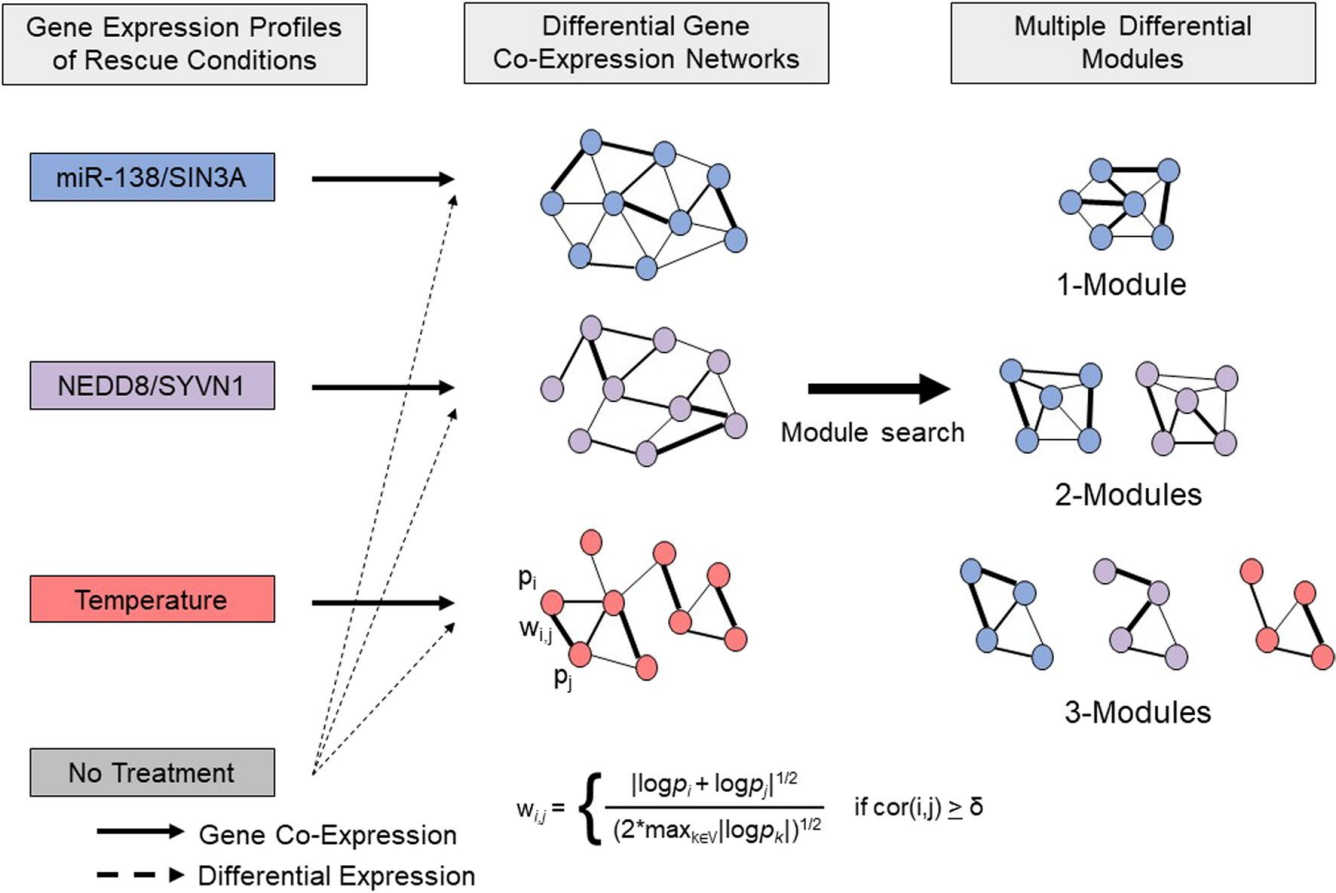
Rescue conditions queried in the M-module framework. Gene expression profiles across the miR-138/SIN3A, NEDD8/SYVN1, and Temperature rescue conditions used to build differential gene co-expression networks. A binary co-expression network was constructed in which genes were selected based on the absolute value of Pearson correlation of the expression profiles of two genes. Only edges whose correlation exceeded a pre-defined threshold were included. Edges were then weighted (**w**_***i,j***_) based on the *p* values (**p**_***i***_ and **p**_***j***_) of differential gene expression between the baseline and rescue conditions. Multiple differential co-expression networks were then analyzed to identify shared and unique multiple differential modules. 1-Modules are unique to a single rescue condition, whereas 2-Modules are found across two rescue conditions, and 3-Modules are found in all three rescue conditions. Adapted from [24]

As shown in Fig. 1, the miR-138, NEDD8, and temperature co-expression profiles were used to create differential networks that yielded multiple M-Modules. Static 1-Modules were unique to an individual rescue condition, where 2-Modules were modules found across two networks and 3-Modules were modules found across all networks. As shown in Table 2, 70 1-Modules containing 964 significant genes were identified in the miR-138 network. The NEDD8 network yielded 55 1-Modules containing 764 significant genes. 44 1-Modules containing 342 significant genes were found in the temperature network. The miR-138 network shared 13 2-Modules containing 159 significant genes with the NEDD8 network and 19 2-Modules containing 191 significant genes with the temperature network. The NEDD8 and temperature networks shared 10 2-Modules containing 160 significant genes. All rescue conditions shared seven 3-Modules containing 103 significant genes.

**Table 2:** M-module query results

Type of module	Condition	# of modules	# of unique genes	# of significant genes (FDR < 0.01)
1-DM	miR-138	70	3109	964
	NEDD8	55	2438	764
	Temperature	44	1433	342
2-DM	miR-138 + NEDD8	13	393	159
	miR-138 + Temperature	19	654	191
	NEDD8 + Temperature	10	305	160
3-DM	miR-138 + NEDD8 + Temperature	7	311	103

### Gene ontology analysis of non-seed module genes identifies enriched CF-relevant terms

**Fig. 2:**
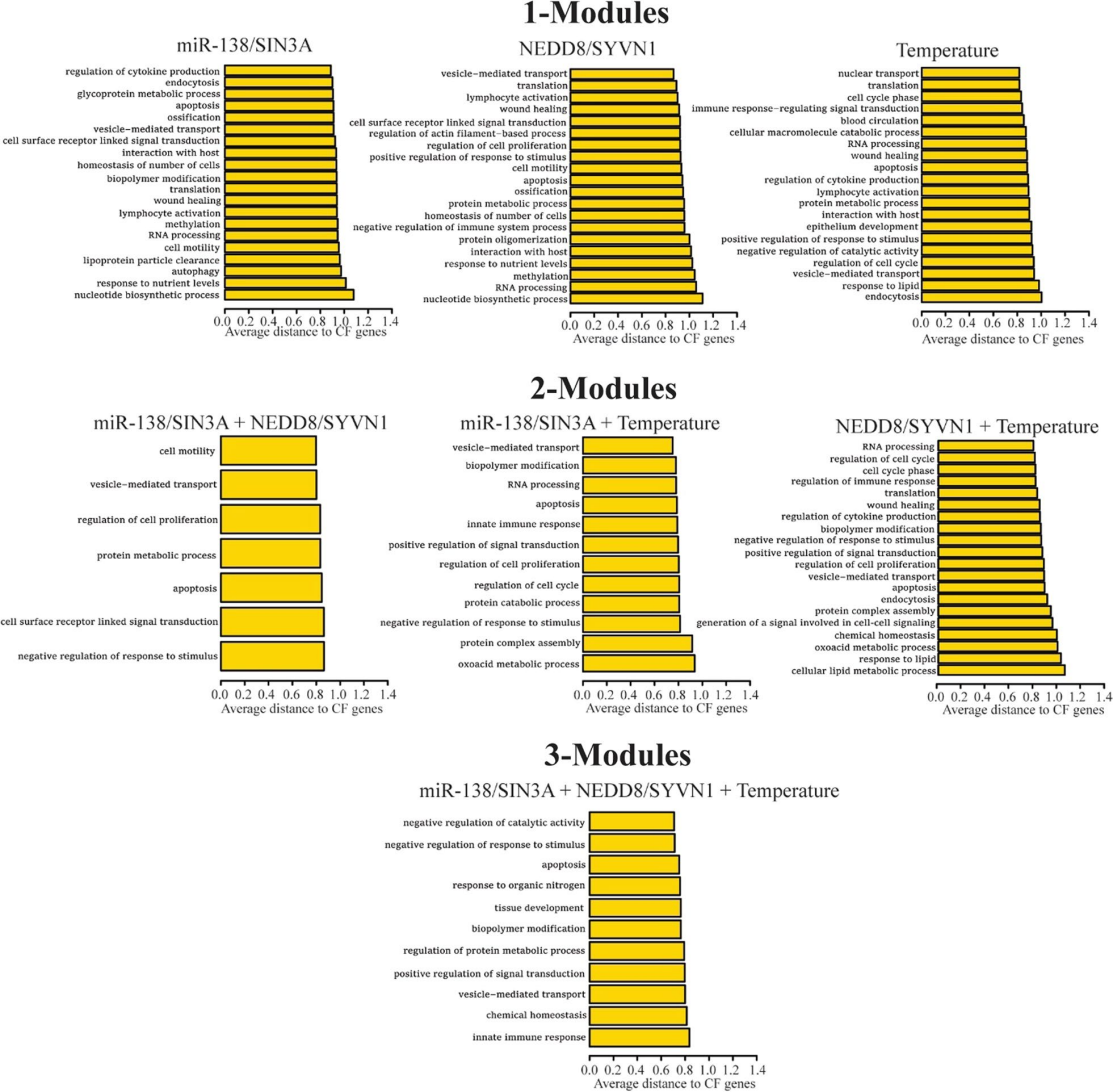
Gene Ontology analysis of non-seed module genes. Enriched terms for significant non-seed module genes found exclusively in 1-Differential Modules (DMs) (top panel), 2-DMs (middle panel), or 3-DMs (bottom panel). X-axis represents the average distance of significant non-seed module genes to CF seed genes

We ranked non-seed significant module genes by minimum distance (the sum of weight of the shortest path between two nodes) to closest seed genes. Gene Ontology (GO) enrichment analysis was performed on the non-seed significant module genes. For each enriched GO term, the average minimum distance between contributing non-seed module genes and CFTR-associated seed genes was calculated. Several CFTR-relevant pathways were identified using this unbiased approach, further validating that M-module could connect genes previously unknown to affect CFTR biogenesis to ΔF508-CFTR-related functions. For example, the following terms were enriched in at least two conditions: translation, protein metabolic/catabolic processes, protein complex assembly, endocytosis, vesicle-mediated transport, apoptosis, and autophagy, among others (Fig. 2).

### Identification of candidate CFTR effectors

**Fig. 3:**
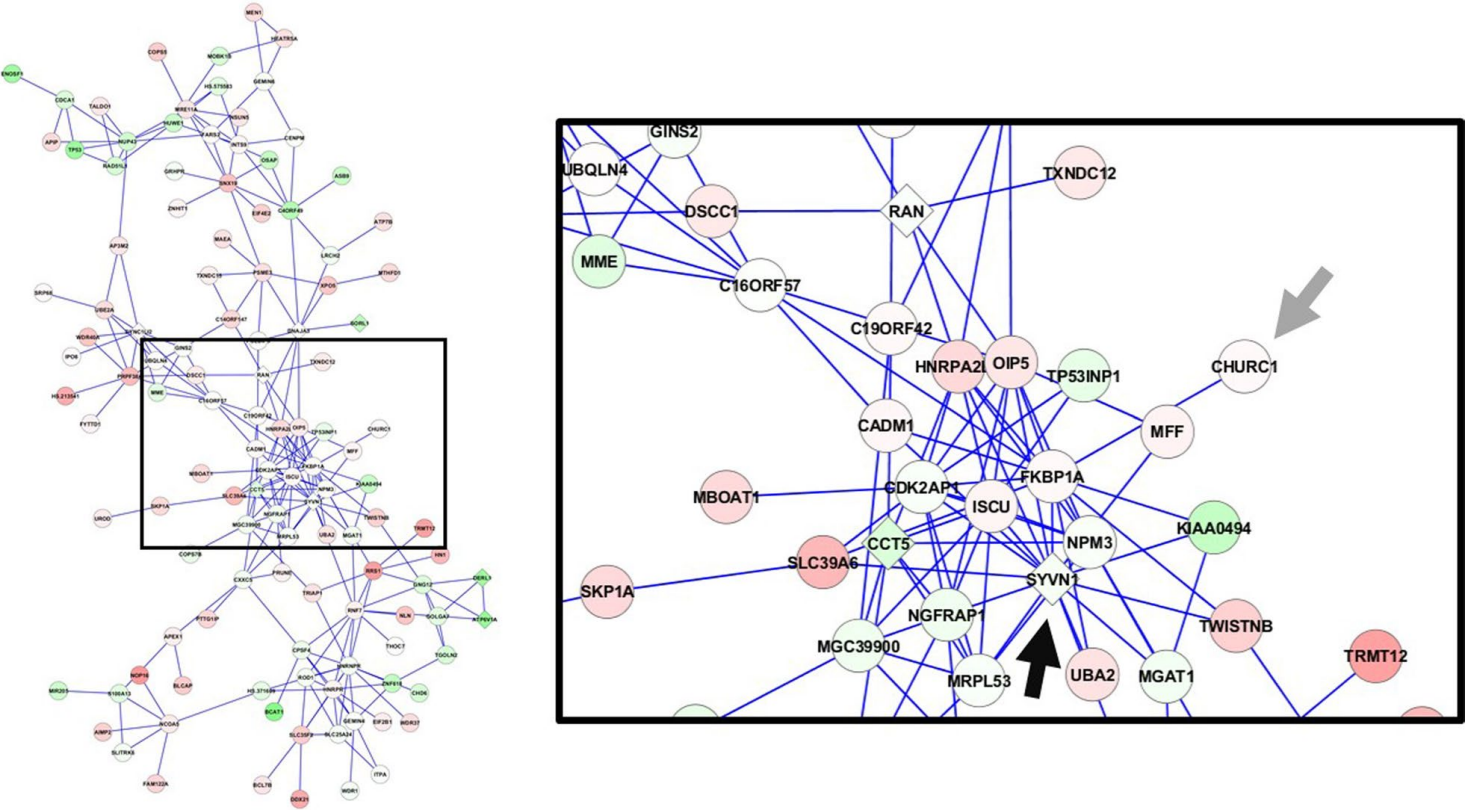
Example 1-Module containing *CHURC1*. The miR-138/SIN3A differential gene co-expression network yielded 70 unique 1-modules, including the 1-Module shown here. Diamonds represent seed genes, while circles signify non-seed genes. The node color is proportional to the −log10 *p* value of gene expression, with red indicating up-regulation and green down-regulation. The inset contains *CHURC1* (gray arrow) and shows that its nearest seed neighbor is *SYVN1* (black arrow). This figure was created using Cytoscape [83]

To identify novel CFTR effectors, significance was first determined by comparing individual module genes to a randomized network. Genes with a FDR < 0.01 were considered significant, narrowing the number of genes across all modules and conditions from 8643 to 2683. Next, all CFTR interactome genes were removed, as were all genes that had previously been tested in various studies, leaving 901 untested non-seed module genes (Additional file 1: Table S7) [18, 19, 35–47]. Due to the known interactions between CFTR and several E3 ubiquitin ligases, we chose to investigate *ASB6* and *ASB13* (components of the SOCS-box ubiquitin ligase complex)*, FBXO46* (F-box)*, ZFAND5* (zf-A20), and the BTB domain-containing proteins *GZF1, KLHL29*, and *ZBTB38* [19, 36, 37, 48–62]. As Lukacs and colleagues recently identified several components of the ribosomal stalk as being CFTR effectors, we also elected to study *RPL15, RPL28,* and *RPL39L* [63]. Lastly, we tested non-seed module genes whose closest seed neighbor was the ERAD-associated protein SYVN1, as our previous studies demonstrated that *SYVN1* knockdown restored partial function to ΔF508-CFTR [19]. These genes included *C11ORF1*, *CADM1*, *CHURC1*, *JPT1*, *MFF*, *POLR1F, RRS1*, and *THOC7*. Figure 3 displays a 1-Module containing *CHURC1* and the neighboring *SYVN1*.

### siRNA-mediated knockdown of CHURC1, GZF1, and RPL15 restores partial function to ΔF508-CFTR

**Fig. 4:**
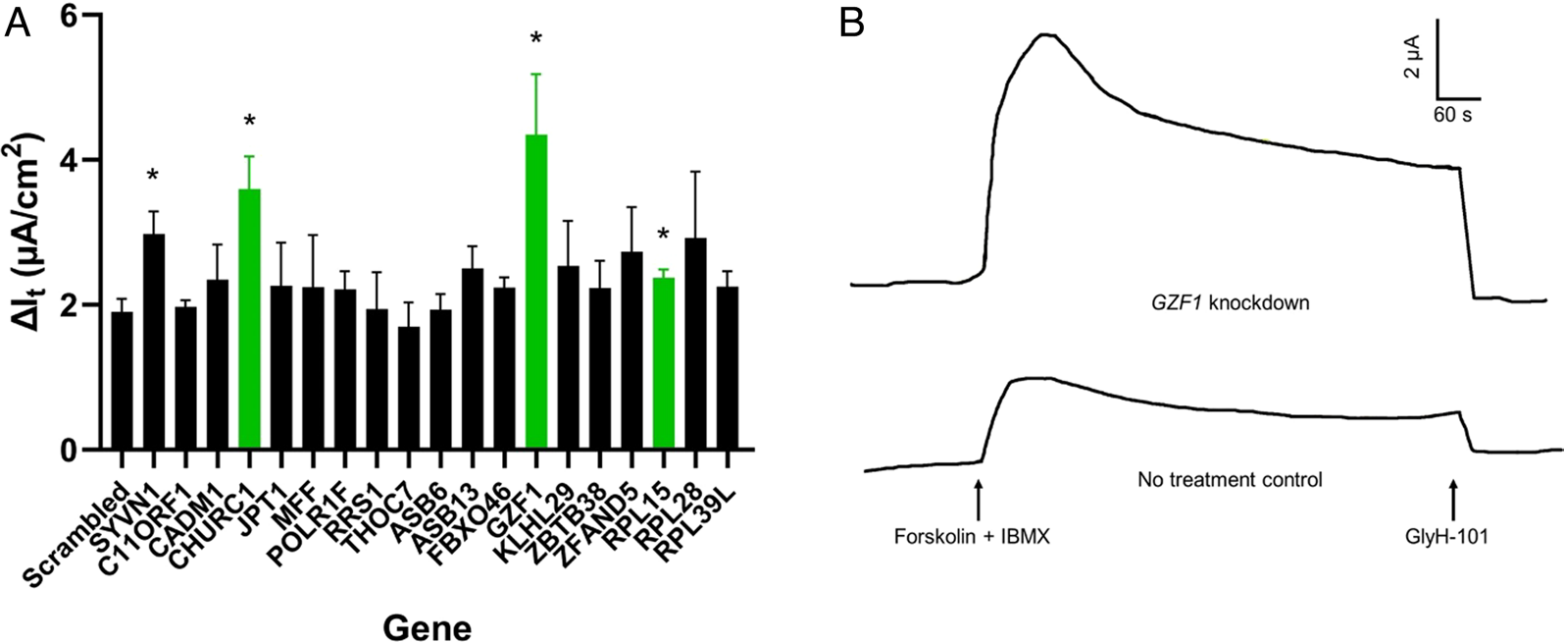
siRNA-mediated knockdown of *CHURC1*, *GZF1*, and *RPL15* rescues ΔF508-CFTR function in CFBE cells. **a** Average change in transepithelial current (I_t_) in response to the cAMP agonists forskolin and IBMX (F&I) and the CFTR inhibitor GlyH-101 under open circuit conditions was measured in CFBE cells. Three siRNAs per gene were pooled and CFBE cells were reverse-transfected using Lipofectamine RNAiMAX and grown on microporous Transwell membranes seven days prior to the electrophysiology measurements. Error bars indicate standard error. Statistical significance compared to the scrambled control was determined by Brown-Forsythe ANOVA and post-hoc Benjamini–Hochberg multiple testing correction (*p < 0.05). n = 4–6 per gene. *CHURC1, GZF1,* and *RPL15* are highlighted in green. **b** Representative transepithelial current tracings showing CFTR-dependent chloride current in CFBE cells treated with a scrambled control or siRNA targeting *GZF1*. The Y-axis represents transepithelial current in µA and the X-axis represents time in seconds

We used pools of three siRNAs per gene to knockdown candidate gene mRNA transcripts in CFBE cells, using the change in cAMP-activated chloride secretion as an endpoint [33]. Knockdown of *CHURC1* significantly improved CFTR-dependent transepithelial chloride current in ΔF508-CFTR CFBE cells, with an increase of 89% in peak chloride current after the addition of forskolin and IBMX compared to the scrambled control (*p* = 0.0112; Fig. 4). Compared to knockdown of *SYVN1, CHURCH1* knockdown increased peak chloride current by over 20%. Knockdown of *GZF1* resulted in an increase of 129% in peak chloride current when compared to the scrambled control (*p* = 0.0405) and an increase of 46% compared to *SYVN1*. Lastly, knockdown of *RPL15* also produced a statistically significant ~ 25% increase in cAMP-activated, GlyH-101-sensitive transepithelial chloride current compared to the scrambled control (*p* = 0.0446). Representative transepithelial current tracings showing CFTR-dependent chloride current in CFBE cells treated with a scrambled control or siRNA targeting *GZF1* are shown in Fig. 4b. Additional representative tracings of knockdown of *CHURCH1*, *RPL15*, and *THOC7* are displayed in Additional file 1: Fig. S2.

## Discussion

While several transcriptomics-based studies have identified effectors, CFTR biogenesis remains incompletely understood. The analysis of the underlying molecular events responsible for ΔF508-CFTR rescue is crucial for understanding the CFTR biosynthetic process and the development of therapeutics. As many transcriptomic and network biology studies of CF focus on diseased versus healthy cells, the resulting seed genes, subnetworks, and edge sets are significantly associated with only one condition and this may limit the detection of CFTR interactors. To address critical gaps in the knowledge of CFTR biogenesis, we applied the M-module framework to reveal subnetwork dynamics by joint analysis of multiple co-expression networks representing ΔF508-CFTR rescue. These networks included miR-138 overexpression and *SIN3A* knockdown, *NEDD8* and *SYVN1* knockdown, and low temperature treatments.

As displayed in Table 2, dozens of unique static modules were identified in the individual conditions, as well as many dynamic modules that were shared between two or all conditions. To assess the quality of these modules, we performed gene ontology (GO) analyses on all significant non-seed module genes across all conditions. Removing all CF-related genes allowed for an unbiased assessment of the functional gene groups represented by the modules. Rather than CF-related genes pushing the gene ontology analysis toward enrichment of CF-related terms, we could instead assess GO enrichments driven exclusively by genes previously unassociated with CFTR. As shown in Fig. 2, many GO enriched terms were related to CF and multiple terms were present across several conditions. For example, genes involved in translation, protein metabolic/catabolic processes, and protein complex assembly were enriched in multiple conditions and both 1- and 2-Modules. As ΔF508-CFTR is classified as a protein folding defect, it is perhaps not surprising that signatures of its rescue were highly enriched for genes involved in translation and protein metabolism [16, 17]. However, the removal of all CFTR-related genes prior to gene ontology analysis means that only genes previously unlinked to CFTR biogenesis were responsible for such GO enrichments. Therefore, is it likely that our modules contain previously undiscovered CFTR effectors that contribute to ΔF508-CFTR rescue.

Likewise, GO analysis indicated that our non-seed modules were enriched for genes involved in endocytosis, vesicle-mediated transport, autophagy, and apoptosis. CFTR is regulated in part by cAMP-dependent vesicle traffic to the apical membrane, although ΔF508-CFTR is typically degraded before reaching the cell surface [64]. Even if mutant protein reaches the cell membrane, clathrin-mediated endocytosis efficiently removes it from the cell surface via recycling endosomes and the protein is directed to lysosomes for degradation [65, 66]. Furthermore, ΔF508-CFTR causes dysfunction of apoptotic and autophagic processes, which may contribute to CF lung disease progression [67–69]. These gene ontology results strongly support the likelihood of our non-seed module genes being members of the CFTR interactome and contributing to its biogenesis.

As our gene ontology analyses suggested that non-seed module genes may contribute to ΔF508-CFTR rescue, we aimed to identify specific genes with such functions. By focusing on genes with *SYVN1* as their nearest seed neighbor, we hypothesized that *CHURC1*, among several other genes, could affect CFTR rescue. siRNA-mediated knockdown of *CHURC1* rescued CFTR-dependent transepithelial chloride current to ΔF508-CFTR by 89% compared to a scrambled control. Knockdown of *CHURC1* even produced an increase of over 20% in peak chloride current when compared to knockdown of *SYVN1*. *CHURC1* (*Churchill Domain Containing 1*) is a transcriptional activator that mediates fibroblast growth factor (FGF) signaling during neural development in zebrafish [70–73]. Rotin and colleagues recently identified FGF Receptor 1 (FGFR1) as a suppressor of ΔF508-CFTR maturation [46]. Further analysis revealed that knockdown of FGFR1, FGFR2, FGFR3, and downstream signaling proteins ERK1, ERK2, AKT, PLCγ-1, and FRS2α, increased ΔF508-CFTR channel activity and protein maturation. These data suggest that *CHURC1* knockdown may rescue mutant CFTR function through the FGF signaling pathway.

To further elucidate potential mechanisms of *CHURC1*, we queried the gene in ARChS^4^, which allows for massive mining of publicly available RNA-seq data [74]. Interestingly, the highest ranked predicted GO biological process for *CHURC1* was ribosomal small subunit biogenesis (Additional file 1: Table S8). Additional enriched terms included cotranslational protein targeting to membrane, protein targeting to ER, translational elongation, ribosomal large subunit biogenesis, translational initiation, and protein complex assembly. Lukacs and colleagues recently identified a component of the ribosomal stalk, *RPL12*, as an effector of ΔF508-CFTR [63]. Silencing of *RPL12* slowed the rate of translation, while increasing the folding efficiency and conformational stability of ΔF508-CFTR. Furthermore, *RPL12* knockdown in combination with lumacaftor restored ΔF508-CFTR function to approximately 50% of the wild-type channel in primary human airway epithelial cells. Lukacs and colleagues also observed that silencing of ribosome stalk proteins RPLP0, RPLP1, and RPLP2 partially rescued ΔF508-CFTR function. In our siRNA knockdown screen, knockdown of *RPL15*, also a protein member of the ribosomal stalk, significantly restored CFTR-dependent chloride secretion. Similar to *CHURC1*, the GO analysis results for *RPL15* were enriched for ribosome-related processes (Additional file 1: Table S9). While *CHURC1* was identified in all three 1-Module conditions, *RPL15* was identified in the NEDD8 and Temperature 1-Modules. Our M-module analysis not only identified *CHURC1* and *RPL15* as potential effectors of CFTR rescue, it also suggests that an underlying mechanism of our queried rescue signatures is the manipulation of translation and the ribosome.

Lending additional support to the candidacy of *CHURC1* as a CFTR effector is the Encyclopedia of DNA Elements (ENCODE), which provides evidence for *SIN3A* transcription factor binding at the promotor of *CHURC1* in several cell types, including the A549 respiratory epithelial cell line [75, 76]. SIN3A can also bind the promoter of *RPL15* in A549 cells, according to ENCODE. Furthermore, TargetScan predicts that miR-138 regulates expression of *CHURC1* [77]. Therefore, it is also possible that *CHURC1* and *RPL15* act through the miR-138/SIN3A pathway we previously described [18]. While GO analysis of *GZF1* did not yield ribosomal- or CFTR-related biological processes (Additional file 1: Table S10), ENCODE and ChIP Enrichment Analysis (ChEA) Transcription Factor Targets both indicate that FOXA1 and FOXA2 bind the promoter of *GZF1* [78, 79]. Harris and colleagues demonstrated that depletion of FOXA1 and FOXA2 represses CFTR expression. Interestingly, these forkhead box transcription factors have also been shown to act as transcriptional repressors [80, 81]. *GZF1* was identified in a miR-138/SIN3A 1-Module and ENCODE also suggests that SIN3A binds the promoter of *GZF1*, supporting the hypothesis that *GZF1* may also act through the miR-138/SIN3A pathway.

## Conclusion

The ability of the M-module to identify dynamic modules involved in ΔF508-CFTR rescue provided a novel approach for studying CFTR biogenesis and allowed for the identification of previously unknown CFTR effectors. siRNA-mediated knockdown of *CHURC1*, *GZF1*, and *RPL15* significantly restored CFTR-dependent transepithelial chloride current in ΔF508-CFTR CFBE cells. Further analysis of these genes and their roles in ΔF508-CFTR rescue and CFTR biogenesis will be required to elucidate exact mechanisms of action.

## Supplementary Information

**Additional file 1**. **Table S1**. Summary of differentially expressed genes across conditions in CFBE cells. Summary of up- and down-regulated differentially expressed genes in conditions vs. controls. **Table S2**. Differentially expressed genes in the miR-138/SIN3A condition. List of differentially expressed genes in the miR-138/SIN3A conditions vs. scrambled siRNA control. **Table S3**. Differentially expressed genes in the NEDD8/SYVN1 condition. List of differentially expressed genes in the NEDD8/SYVN1 conditions vs. scrambled siRNA control. **Table S4**. Differentially expressed genes in the temperature condition. List of differentially expressed genes in the temperature conditions vs. 37°C control. **Table S5**. CFTR interactome used as seed nodes. List of CFTR effectors and interactors used as seed nodes in the M-module analysis. **Table S6**. DsiRNA and primer sequences. List of siRNA and primer sequences used in the functional knockdown experiments to test for CFTR rescue. **Table S7**. Untested non-seed module genes. List of genes resulting from the M-module analysis that have not been previously tested or linked to CFTR. **Table S8**. Top 50 predicted gene ontology biological processes for *CHURC1*. List of biological processes associated with *CHURC1* according to the ARChS^4^ software. **Table S9**. Top 50 predicted gene ontology biological processes for *RPL15*. List of biological processes associated with *RPL15* according to the ARChS^4^ software. **Table S10**. Top 50 predicted gene ontology biological processes for *GZF1*. List of biological processes associated with *GZF1* according to the ARChS^4^ software. **Figure S1**. Schematic showing intersection of differentially expressed genes across conditions. Controls and conditions are described in Table 1. Up arrows indicate up-regulated genes; down arrows indicate down-regulated genes. Significance is defined as FDR < 0.05. **Figure S2**. Representative transepithelial current tracings demonstrating the effects of individual gene knockdown on CFTR-dependent chloride current in CFBE cells. The Y-axis represents transepithelial current in µA and the X-axis represents time in seconds. The addition of the cAMP agonists forskolin and IBMX resulted in an increase in CFTR-dependent transepithelial chloride current in cells treated with DsiRNAs targeting: A) CHURC1 or B) RPL15. This increase in current was inhibited by the CFTR channel inhibitor GlyH-101. The tracing shown in C demonstrates that DsiRNA knockdown of THOC7 was ineffective in restoring CFTR-dependent chloride current.

## Data Availability

The datasets analyzed during the current study are available in the Gene Expression Omnibus Series GSE142610 (https://www.ncbi.nlm.nih.gov/geo/query/acc.cgi?acc=GSE142610) [82]. M-module software can be installed at https://github.com/tanlabcode/M-module. Individual modules and datasets are available from the corresponding author on reasonable request.

## Abbreviations

CF: Cystic fibrosis
CFBE: Cystic fibrosis bronchial epithelia
CFTR: Cystic fibrosis transmembrane conductance regulator
DIDS: 4,4′-Diisothiocyanoto-stilbene-2,2′-disulfonic acid
DsiRNA: Dicer-substrate short interfering RNA
ERAD: Endoplasmic reticulum-associated degradation
GO: Gene ontology
IBMX: 3-Isobutyl-1-methylxanthine
